# A study on the spectral properties of covariance type matrices with isotropic log-concave column vectors

**DOI:** 10.1371/journal.pone.0297150

**Published:** 2024-01-26

**Authors:** Shaojia Jin, Zhanwen Shi

**Affiliations:** 1 School of Mathematical and Physical Sciences, Wuhan Textile University, Wuhan, China; 2 School of Business Administration, Zhongnan University of Economics and Law, Wuhan, China; Federal University of Pernambuco: Universidade Federal de Pernambuco, BRAZIL

## Abstract

The limiting spectral distribution of matrix $B_{n}=\frac{1}{N}X_{n}X_{n}^{*}T_{n}$ is considered in this paper. Existing results always focus on the condition of modifying *T*_*n*_, but for *X*_*n*_, it is usually assumed to be a matrix composed of *n* × *N* independent identically distributed elements. Here we specify the joint distribution of column vectors of *X*_*n*_. In particular, entries on the same column of *X*_*n*_ are correlated, in contrast with more common independence assumptions. Assuming that the columns of *X*_*n*_ are random vectors following the isotropic log-concave distribution, and under some additional regularity conditions, we prove that the empirical spectral distribution $F^{B_{n}}$ of matrix *B*_*n*_ converges to a deterministic probability distribution *F* almost surely. Moreover, the Stieltjes transformation *m* = *m*(*z*) of *F* satisfies a deterministic form of equation, and for any $z\in C^{+}$, it is the unique solution of the equation.

## 1. Introduction

In multivariate statistical analysis, the distribution of the eigenvalues of a random matrix is widely applied to test various hypotheses. The distribution is also used in nuclear physics, since the behavior of highly excited energy levels in nuclear physics can be explained by considering the distribution of eigenvalues of some random matrices. For any *n* × *n* square matrix *A*_*n*_ with only real eigenvalues denoted by λ_1_, λ_2_, …, λ_*n*_, the empirical spectral distribution function of *A*_*n*_ can be defined as

$$\begin{array}{c} F^{A_{n}}\left(x\right)=\frac{1}{n}\sum_{i=1}^{n}I_{\left\{\lambda_{i}\leq x\right\}}, \end{array}$$

where *I*_*A*_ denote the indicator function. If in the large *n* limit the limiting distribution $F^{A_{n}}\left(x\right)$ exists, then this limit is referred to as the limiting spectral distribution of *A*_*n*_. The limiting spectral properties of large random matrices have aroused great interests in statistics, finance, signal processing, and other disciplines. Interested readers can refer to Bai and Silverstein [1] to make a comprehensive review of this rapidly developing research field.

This paper analyzes the spectral property of the product of random matrices, which is useful for many problems in multivariate statistical analysis. For example, in multivariate discriminant analysis of variance analysis, and test the equality of two covariance matrices when the potential distribution is multivariate normal, it is interesting to consider the eigenvalues of a multivariate *F* matrix, where *F* is known to be the central multivariate *F* matrix when the two random matrices are distributed independent of each other as central Wishart matrices. Considerable works are conducted in the past on the theory of asymptotic distributions of certain functions of the eigenvalues of the multivariate *F* matrix, which is basically limited to the case where the number of variables is fixed and the sample size tends to infinity. However, many situations arise when the experimenter is confronted with the problem of inferring from the data with a very large number of variables. Here, we consider the general *F* matrix $B_{n}=\frac{1}{N}X_{n}X_{n}^{*}T_{n}$, which reduces to the central multivariate *F* matrix when *T*_*n*_ is the inverse of another independent covariance matrix. For the special case of *T* = *I* = identity, there are also many results available, see Grenader and Silverstein [2], Wachter [3], as well as Yin and Krishnaiah [4].

For random matrix $B_{n}=\frac{1}{N}X_{n}X_{n}^{*}T_{n}$, which is also a model often involved in the wireless communication, we focus on the limiting spectral distribution of *X*_*n*_, when the column vectors of *X*_*n*_ are isotropic log-concave distributed in this paper. That is to say, the collected signal data is considered to be a whole vector, rather than composes of many unrelated signal components. As is well known, the isotropic logarithmic concave distribution has also been a widely studied distribution type in the fields of economics, statistics, and information theory in recent years [5]. By the way, the studies of randomness and matrices have always also been a topic of great concern for many other professional researchers (see [6–9]). In fact, any non-degenerate zero mean random vector can become an isotropic random vector after the proper linear transformation, such as the standard normal distribution random vector, Bernoulli type random vector with independent Bernoulli random variable components, and uniformly distributed random vector on convex set in *R*_*n*_.

The research on the model $B_{n}=\frac{1}{N}X_{n}X_{n}^{*}T_{n}$ starts from a class of important Hermitian random matrices with the form of ${\underline{B}}_{n}=\frac{1}{N}X_{n}^{*}T_{n}X_{n}$, where matrix *X*_*n*_ = (*X*_*ij*_)_*n*×*N*_ is a complex random matrix, and random variables *X*_*ij*_’s are independent identically distributed (i.i.d.), where *T*_*n*_ is a *n* × *n* diagonal matrix and is independent of *X*_*n*_, and its empirical spectral distribution $F^{T_{n}}\overset{d}{\to }H,\;a.s.$, where *H* is a non-random probability distribution defined on [0, ∞). With regard to ${\underline{B}}_{n}$, the earliest researches are focused by Marčenko and Pastur [10] and Grenander and Silverstein [2], who proved that when *n*, *N* → ∞ and $\frac{n}{N}\to c$, the empirical spectral distribution $F^{{\underline{B}}_{n}}$ of matrix ${\underline{B}}_{n}$ weakly converges to a non-random probability distribution in probability. Then, Wachter [3], Jonsson [11], Yin [12] and Yin and Krishnaiah [4] conducted further research, and illustrated that the empirical spectral distribution $F^{{\underline{B}}_{n}}$ converges weakly to a certain non-random probability distribution almost surely. It is noted that Yin and Krishnaiah [4] and Yin [12] established this result under the assumption that matrix *T*_*n*_ is not a diagonal matrix, but a Hermitian nonnegative definite matrix. Moreover, Yin [12] weakened the condition for *X*_*n*_, while other results required that the element moment condition of *X*_*n*_ is at least higher than the second order. Except for Marčenko and Pastur [10], others only studied the case under the assumption that the element of *X*_*n*_ is real. More importantly, only Marčenko and Pastur [10] considered the case where *H* is arbitrary, while others require *H* to satisfy all moment conditions and adopt moment methods to prove the limit theorem.

Based on Yin’s results [12], Silverstein [13] studied the matrix $B_{n}=\frac{1}{N}X_{n}X_{n}^{*}T_{n}$ by generalizing Yin’s assumptions [12], and extended the condition that “*X*_*ij*_’s are real random variables” to complex cases, and studied the behavior when *H* is arbitrary. It was proved by Stieltjes transformation method that, almost surely, the empirical spectral distribution of $B_{n}=\frac{1}{N}X_{n}X_{n}^{*}T_{n}$ converges weakly to a non-random probability distribution. Silverstein and Bai [14] found that when matrix *T*_*n*_ is an arbitrary diagonal matrix, the empirical spectral distribution of $F^{\frac{1}{N}X_{n}^{*}T_{n}X_{n}}\left(x\right)\overset{d}{\to }\overset{`}{F},\;x\neq 0,\;a.s.$, where $\overset{`}{F}$ denotes a non-random probability distribution. Let $m_{\overset{`}{F}}$ be the Stieltjes transformation of $\overset{`}{F}$, then $m_{\overset{`}{F}}$ is the solution of equation

$$\begin{array}{c} m\left(z\right)=-{\left(z-c\int \frac{\tau dH(\tau )}{1+\tau m(z)}\right)}^{-1}. \end{array}$$ (1)

Moreover, for any $z\in C^{+}$, $m=m_{\overset{`}{F}}$ is the unique solution of Eq (1). Assuming that all elements of matrix *X*_*n*_ are i.i.d. random variables, Silverstein [13] investigated matrix $B_{n}=\frac{1}{N}X_{n}X_{n}^{*}T_{n}$ based on the case that matrix *T*_*n*_ is a Hermitian non-negative definite matrix. Comparing the spectra of matrices ${\underline{B}}_{n}=\frac{1}{N}X_{n}^{*}T_{n}X_{n}$ and $B_{n}=\frac{1}{N}X_{n}X_{n}^{*}T_{n}$, we find that there is a difference of |*n* − *N*| zero eigenvalues between them. It is then easy to obtain the relationship between their empirical spectral distribution function, which is given by

$$\begin{array}{c} F^{{\underline{B}}_{n}}=\left(1-c_{n}\right)I_{\left[0,\infty \right)}+c_{n}F^{B_{n}}, \end{array}$$ (2)

where $c_{n}=\frac{n}{N}$. Then, the relationship of Stieltjes transformation of their empirical spectral distribution is

$$\begin{array}{c} m_{F^{{\underline{B}}_{n}}}\left(z\right)=-\frac{\left(1-c_{n}\right)}{z}+c_{n}m_{F^{B_{n}}}\left(z\right),\;\;z\in C^{+}. \end{array}$$ (3)

If there exist the limits of empirical spectral distributions $F^{{\underline{B}}_{n}}$ and $F^{B_{n}}$, they are denoted by ${\overset{`}{F}}^{\prime }$ and *F*, respectively. Then we calculate the limits of Eqs (2) and (3), respectively, and have

$$\begin{array}{c} {\overset{`}{F}}^{\prime }=\left(1-c\right)I_{\left[0,\infty \right)}+cF \end{array}$$

and

$$\begin{array}{c} m_{{\overset{`}{F}}^{\prime }}\left(z\right)=-\frac{\left(1-c\right)}{z}+cm_{F}\left(z\right),\;\;z\in C^{+}. \end{array}$$ (4)

Then, if ${\overset{`}{F}}^{\prime }=\overset{`}{F}$ is conjectured, it can be obtained from Eqs (1) and (4) that the Stieltjes transformation *m* = *m*_*F*_(*z*) of the limiting spectral distribution of matrix $B_{n}=\frac{1}{N}X_{n}X_{n}^{*}T_{n}$ is the solution of equation

$$\begin{array}{c} m\left(z\right)=\int \frac{1}{\tau (1-c-czm(z))-z}dH\left(\tau \right), \end{array}$$ (5)

and the solution is unique on the set $\left\{m\in C:-\frac{\left(1-c\right)}{z}+cm\in C^{+}\right\}$. In this paper, the limiting spectral distribution of such a matrix model is studied: suppose that the column vector *X*_⋅*j*_ of *X*_*n*_, and we specify the joint distribution of column vectors of *X*_*n*_. In particular, entries on the same column of *X*_*n*_ are correlated, in contrast with more common independence assumptions. Assume that *X*_⋅*j*_ is an *n*-dimensional random vector following isotropic log-concave distribution. In the following, we continue to focus on the case of matrix model $B_{n}=\frac{1}{N}X_{n}X_{n}^{*}T_{n}$ with i.i.d. column vector *X*_⋅*j*_ of *X*_*n*_ being isotropic log-concave distributed. Here are the corresponding definitions.

**Definition 1.1**. *A random real vector* $X\in R^{n}$ *follows isotropic distribution, if it satisfies*

$$E\left\langle X,y\right\rangle ={0,\;\;\;E|\left\langle X,y\right\rangle |}^{2}={\left\|y\right\|}^{2},\;\;\forall \;y\in R^{n},$$

*where* 〈, 〉 *is the ordinary inner product, and* ‖⋅‖ *is ordinary Euclidean norm*. *A random complex vector* $X\in C^{n}$ *follows isotropic distribution, if* $\left(ReX,ImX\right)\in R^{2n}$ *follows isotropic distribution, and* *ReX* *is independent of* *ImX*, *where* *ReX and ImX represent the real part and the imaginary part of X, respectively*.

**Remark 1.1**. *According to the above definition, if X follows isotropic distribution, then EX* = 0, Σ(*X*) = *cov*(*X*, *X*) = *I*_*n*_. *Any non-degenerate random vector can be transformed into a random vector obeying isotropic distribution by appropriate linear transformation. In fact, if the mean value of any random vector* $X\in R^{n}$ *is* 0, *and its covariance matrix* Σ(*X*) *is reversible, then* ${\left(\Sigma \left(X\right)\right)}^{\frac{1}{2}}X$ *follows isotropic distribution*.

**Definition 1.2**. *A function* $f:\;R^{n}\to R$ *is log-concave, if for any* $x_{1},x_{2}\in R,\;\theta \in \left[0,1\right]$,

$$\begin{array}{c} f\left(\theta x_{1}+\left(1-\theta \right)x_{2}\right)\geq f{\left(x_{1}\right)}^{\theta }f{\left(x_{2}\right)}^{1-\theta }. \end{array}$$

**Remark 1.2**. *If X is an n-dimensional continuous random variable with a log-concave distribution, and its density function is f*(*x*), *the marginal density function corresponding to any of its sub-vectors also follows a log-concave distribution. And if X follows the log-concave distribution, then any affine transformation of X follows the log-concave distribution. Log-concave distribution constitutes a large class of distributions, such as multidimensional uniform distribution, multidimensional normal distribution, multidimensional exponential distribution, etc*.

Prior to giving the main results, we introduce some notations. $X_{n}\overset{d}{\to }X$ implies that a random variable sequence {*X*_*n*_} converges to the random variable *X* in distribution. Denote the trace of matrix *A* by *tr*(*A*). Let *E* be the expectation of matrix or vector, and *C* be a positive constant, and its value may be different in the context. Now, we present the main results.

**Theorem 1.1**. *Suppose that*

*(a)* *X*_*n*_ *is an n* × *N complex random matrix, and the column vector X*_⋅*j*_ (*j* = 1, ⋯, *N*) *is a random vector with n-dimensional mutually independent isotropic log-concave distribution* [15, 16].

*(b)* *T*_*n*_ *is an n* × *n Hermitian non-negative definite matrix, and when n* → ∞, *its empirical spectral distribution* $F^{T_{n}}\overset{d}{\to }H,\;a.s.$, *where H is any probability distribution defined on* [0, ∞).

*(c) T*_*n*_ *is independent of X*_*n*_.

*(d) When n*, *N* → ∞, $\frac{n}{N}\to c$.

*Then, the empirical spectral distribution* $F^{B_{n}}$ *of matrix B*_*n*_ *converges weakly to a non-random distribution F almost surely, and the Stieltjes transformation m* = *m*(*z*) *of F satisfies the* Eq (5) *for any* $z\in C^{+}$. *And on* $D_{c}=\left\{m\in C:-\frac{\left(1-c\right)}{z}+cm\in C^{+}\right\}$, *m* = *m*(*z*) *is the unique solution of* Eq (5).

## 2. Proofs

In the proofs, we mainly use the continuity theorem of Stieltjes transform and the properties of isotropic log-concave random variables. Recall that, the Stieltjes transform of a distribution function *F*(*x*) is given by

$$\begin{array}{c} s_{F}\left(z\right)=\int \frac{1}{x-z}dF\left(x\right),\;z\in C^{+}=\left\{z=u+iv|u\in R,v>0\right\}. \end{array}$$

Then, we can write the Stieltjes transform of empirical spectral distribution $F^{A_{n}}\left(x\right)$ as

$$\begin{array}{c} s_{F^{A_{n}}}\left(z\right)=\frac{1}{n}\sum_{i=1}^{n}\frac{1}{\lambda_{i}-z}=\frac{1}{n}tr{\left(A_{n}-zI_{n}\right)}^{-1}, \end{array}$$

where *I*_*n*_ denotes the identity matrix of size *n*. For a sequence of functions of bounded variation {*G*_*n*_}, whose Stieltjes transform is $m_{G_{n}}\left(z\right)$, where *G*_*n*_(−∞) = 0 for all *n*, and a function of bounded variation *G*, where *G*(−∞) = 0, whose Stieltjes transform is *m*_*G*_(*z*), then the continuity theorem of Stieltjes transformation shows that *G*_*n*_ converges vaguely to *G* when $m_{G_{n}}\left(z\right)$ converges to *m*_*G*_(*z*) uniformly for $z\in C^{+}$. Since the empirical spectral distribution sequence of random matrix is tight (Lytova and Pastur [17]), we can use the convergence of its corresponding Stieltjes transformation to obtain the weak convergence of empirical spectral distribution. In addition, according to the almost surely convergence of Stieltjes transformation, the almost surely convergence of empirical spectral distribution is obtained if the limiting spectral distribution is a deterministic probability density function.

Now, we give the proof of Theorem 1.1. Recall that $B_{n}=\frac{1}{N}X_{n}X_{n}^{*}T_{n}$ and ${\underline{B}}_{n}=\frac{1}{N}X_{n}^{*}T_{n}X_{n}$, and let *m*_*n*_(*z*) and ${\underline{m}}_{n}\left(z\right)$ be Stieltjes transformations of empirical spectral distribution $F^{B_{n}}$ and $F^{{\underline{B}}_{n}}$, respectively. Let $B_{n}=\sum_{j=1}^{N}r_{j}r_{j}^{*}$, where $r_{j}=\frac{1}{\sqrt{N}}T_{n}^{\frac{1}{2}}X_{\cdot j}$, and *X*_⋅*j*_ represents *j*-th column of *X*_*n*_. Then, the proof of Theorem 1.1 is obtained by the following lemmas.

**Lemma 2.1**. *For any fixed* $z=u+iv\in C^{+}$, *let* $B_{j}=B_{n}-r_{j}r_{j}^{*}$, *then*

$$\begin{array}{c} {\underline{m}}_{n}\left(z\right)=-\frac{1}{N}\sum_{j=1}^{N}\frac{1}{z(1+r_{j}^{*}{\left(B_{j}-zI\right)}^{-1}r_{j})}. \end{array}$$

*Proof*. For any $z=u+iv\in C^{+}$,

$$\begin{array}{c} B_{n}-zI+zI=\sum_{j=1}^{N}r_{j}r_{j}^{*}. \end{array}$$

Since (*B*_*n*_ − *zI*) is invertible, we have

$$\begin{array}{c} \frac{1}{N}tr\left(I+z{\left(B_{n}-zI\right)}^{-1}\right)=c_{n}+zc_{n}m_{n}=\frac{1}{N}\sum_{j=1}^{N}r_{j}^{*}{\left(B_{n}-zI\right)}^{-1}r_{j}. \end{array}$$ (6)

For any integer 1 ≤ *j* ≤ *N*, we define

$$B_{j}=B_{n}-r_{j}r_{j}^{*}.$$

From

$${\left(B_{n}-zI\right)}^{-1}-{\left(B_{j}-zI\right)}^{-1}=-{\left(B_{j}-zI\right)}^{-1}r_{j}r_{j}^{*}{\left(B_{n}-zI\right)}^{-1},$$

we know that

$$\begin{array}{c} r_{j}^{*}{\left(B_{n}-zI\right)}^{-1}r_{j}-r_{j}^{*}{\left(B_{j}-zI\right)}^{-1}r_{j}=-r_{j}^{*}{\left(B_{n}-zI\right)}^{-1}r_{j}r_{j}^{*}{\left(B_{j}-zI\right)}^{-1}r_{j}. \end{array}$$

Moreover, we have

$$\begin{array}{c} r_{j}^{*}{\left(B_{n}-zI\right)}^{-1}r_{j}=\frac{1}{1+r_{j}^{*}{\left(B_{j}-zI\right)}^{-1}r_{j}}r_{j}^{*}{\left(B_{j}-zI\right)}^{-1}r_{j}. \end{array}$$ (7)

From (3), we know that

$$\begin{array}{c} z{\underline{m}}_{n}=-1+c_{n}+zc_{n}m_{n}. \end{array}$$ (8)

Then combining (6), (7) and (8), we obtain that

$$\begin{array}{c} {\underline{m}}_{n}\left(z\right)=-\frac{1}{N}\sum_{j=1}^{N}\frac{1}{z(1+r_{j}^{*}{\left(B_{j}-zI\right)}^{-1}r_{j})}. \end{array}$$

The proof of Lemma 2.1 is completed.

**Lemma 2.2**. *For n* × *n* *matrices B and* $q\in C^{n}$, *where B and B* + qq* *are invertible, then*

$$\begin{array}{c} q^{*}{\left(B+qq^{*}\right)}^{-1}=\frac{1}{1+q^{*}B^{-1}q}q^{*}B^{-1}. \end{array}$$ (9)

*Proof*. Let q*(*B* + qq*)^−1^ ≡ t*, then q* = t*B + t*qq*. Multiply *B*^−1^ on both sides of Eq (9), and then we have

$$\begin{array}{c} q^{*}B^{-1}=t^{*}+t^{*}qq^{*}B^{-1}. \end{array}$$ (10)

Multiply *q* on both sides of Eq (10), and we get that

$$q^{*}B^{-1}q=t^{*}q+t^{*}qq^{*}B^{-1}q=t^{*}q\left(1+q^{*}B^{-1}q\right).$$

Because *q* is arbitrary, we obtain that 1 + q*B^−1^*q* ≠ 0. Thus,

$$\begin{array}{c} t^{*}q=\frac{q^{*}B^{-1}q}{1+q^{*}B^{-1}q}. \end{array}$$ (11)

From Eq (10), we obtain that

$$\begin{array}{c} t^{*}=q^{*}B^{-1}-t^{*}qq^{*}B^{-1}. \end{array}$$ (12)

Then, combining (11) and (12), we have

$$\begin{array}{cc} t^{*} & =q^{*}B^{-1}-\frac{q^{*}B^{-1}q}{1+q^{*}B^{-1}q}q^{*}B^{-1}\\ & =\frac{1}{1+q^{*}B^{-1}q}q^{*}B^{-1}. \end{array}$$

Namely,

$$\begin{array}{c} q^{*}{\left(B+qq^{*}\right)}^{-1}=\frac{1}{1+q^{*}B^{-1}q}q^{*}B^{-1}. \end{array}$$

The Eq (9) is proved.

**Lemma 2.3**. *For matrix* $A_{n}=-z{\underline{m}}_{n}\left(z\right)T_{n}$, *we have*

$$\begin{array}{c} \frac{1}{n}tr{\left(A_{n}-zI\right)}^{-1}-m_{n}\left(z\right)=\frac{1}{N}\sum_{j=1}^{N}\frac{-1}{z(1+r_{j}^{*}{\left(B_{j}-zI\right)}^{-1}r_{j})}d_{j}, \end{array}$$

*where*

$$d_{j}=q_{j}^{*}T_{n}^{\frac{1}{2}}{\left(B_{j}-zI\right)}^{-1}{\left({\underline{m}}_{n}\left(z\right)T_{n}+I\right)}^{-1}T_{n}^{\frac{1}{2}}q_{j}-\frac{1}{n}tr{\left({\underline{m}}_{n}\left(z\right)T_{n}+I\right)}^{-1}T_{n}{\left(B_{n}-zI\right)}^{-1}$$

*and*

$$q_{j}=\frac{1}{\sqrt{n}}X_{\cdot j}.$$

*Proof*. Define $A_{n}=-z{\underline{m}}_{n}\left(z\right)T_{n}$, then

$$\begin{array}{c} B_{n}-zI-\left(A_{n}-zI\right)=\sum_{j=1}^{N}r_{j}r_{j}^{*}-\left(-z{\underline{m}}_{n}\left(z\right)\right)T_{n}. \end{array}$$ (13)

By Lemmas 2.1 and 2.2 and Eq (13), we have

$$\begin{array}{cc} & {\left(A_{n}-zI\right)}^{-1}-{\left(B_{n}-zI\right)}^{-1}\\ = & {\left(-z{\underline{m}}_{n}\left(z\right)T_{n}-zI\right)}^{-1}(\sum_{j=1}^{N}r_{j}r_{j}^{*}-\left(-z{\underline{m}}_{n}\left(z\right)\right)T_{n}){\left(B_{n}-zI\right)}^{-1}\\ = & {\left(-z{\underline{m}}_{n}\left(z\right)T_{n}-zI\right)}^{-1}\sum_{j=1}^{N}r_{j}r_{j}^{*}{\left(B_{j}+r_{j}r_{j}^{*}-zI\right)}^{-1}\\ & -{\left(-z{\underline{m}}_{n}\left(z\right)T_{n}-zI\right)}^{-1}\left(-z{\underline{m}}_{n}\left(z\right)\right)T_{n}{\left(B_{n}-zI\right)}^{-1}\\ & \sum_{j=1}^{N}\frac{-1}{z(1+r_{j}^{*}{\left(B_{j}-zI\right)}^{-1}r_{j})}({\left({\underline{m}}_{n}\left(z\right)T_{n}+I\right)}^{-1}r_{j}r_{j}^{*}{\left(B_{j}-zI\right)}^{-1}\\ & -\frac{1}{N}{\left({\underline{m}}_{n}\left(z\right)T_{n}+I\right)}^{-1}T_{n}{\left(B_{n}-zI\right)}^{-1}). \end{array}$$

Take the trace of two sides of the above equation, and divide by *n*, then we get that

$$\begin{array}{c} \frac{1}{n}tr{\left(A_{n}-zI\right)}^{-1}-m_{n}\left(z\right)=\frac{1}{N}\sum_{j=1}^{N}\frac{-1}{z(1+r_{j}^{*}{\left(B_{j}-zI\right)}^{-1}r_{j})}d_{j}, \end{array}$$

where

$$d_{j}=q_{j}^{*}T_{n}^{\frac{1}{2}}{\left(B_{j}-zI\right)}^{-1}{\left({\underline{m}}_{n}\left(z\right)T_{n}+I\right)}^{-1}T_{n}^{\frac{1}{2}}q_{j}-\frac{1}{n}tr{\left({\underline{m}}_{n}\left(z\right)T_{n}+I\right)}^{-1}T_{n}{\left(B_{n}-zI\right)}^{-1},$$

$$q_{j}=\frac{1}{\sqrt{n}}X_{\cdot j}.$$

The proof of Lemma 2.3 is completed.

**Lemma 2.4**. *For matrix* $A_{n}=-z{\underline{m}}_{n}\left(z\right)T_{n}$, *we have*

$$\begin{array}{c} \frac{1}{n}tr{\left(A_{n}-zI\right)}^{-1}-m_{n}\left(z\right)\to 0,\;n\to \infty . \end{array}$$

*Proof*. By Lemma 2.3, we have

$$\begin{array}{c} \frac{1}{n}tr{\left(A_{n}-zI\right)}^{-1}-m_{n}\left(z\right)=\frac{1}{N}\sum_{j=1}^{N}\frac{-1}{z(1+r_{j}^{*}{\left(B_{j}-zI\right)}^{-1}r_{j})}d_{j}. \end{array}$$

For any *z* = *u* + *iv*, and any *j*, we have

$$\begin{array}{cc} & Im(r_{j}^{*}{\left(\frac{1}{z}B_{j}-I\right)}^{-1}r_{j})\\ = & \frac{1}{2i}r_{j}^{*}({\left(\frac{1}{z}B_{j}-I\right)}^{-1}-{\left(\frac{1}{\bar{z}}B_{j}-I\right)}^{-1})r_{j}\\ = & \frac{v}{{\left|z\right|}^{2}}r_{j}^{*}{\left(\frac{1}{z}B_{j}-I\right)}^{-1}B_{j}{\left(\frac{1}{\bar{z}}B_{j}-I\right)}^{-1}r_{j}\\ \geq & 0. \end{array}$$

Thus,

$$\begin{array}{c} |\frac{1}{N}\sum_{j=1}^{N}\frac{1}{z(1+r_{j}^{*}{\left(B_{j}-zI\right)}^{-1}r_{j})}|\leq \frac{1}{v}. \end{array}$$

Then for *d*_*j*_, we have

$$\begin{array}{cc} & E|q_{j}^{*}T_{n}^{\frac{1}{2}}{\left(B_{j}-zI\right)}^{-1}{\left({\underline{m}}_{n}\left(z\right)T_{n}+I\right)}^{-1}T_{n}^{\frac{1}{2}}q_{j}-\frac{1}{n}tr{\left({\underline{m}}_{n}\left(z\right)T_{n}+I\right)}^{-1}T_{n}{\left(B_{n}-zI\right)}^{-1}{|}^{2}\\ \leq & \frac{1}{n^{2}}(E|X_{\cdot j}^{*}T_{n}^{\frac{1}{2}}{\left(B_{j}-zI\right)}^{-1}{\left({\underline{m}}_{n}\left(z\right)T_{n}+I\right)}^{-1}T_{n}^{\frac{1}{2}}X_{\cdot j}-tr{\left({\underline{m}}_{n}\left(z\right)T_{n}+I\right)}^{-1}\\ & T_{n}{\left(B_{j}-zI\right)}^{-1}{|}^{2}+E|tr{\left({\underline{m}}_{n}\left(z\right)T_{n}+I\right)}^{-1}T_{n}{\left(B_{j}-zI\right)}^{-1}-tr{\left({\underline{m}}_{n}\left(z\right)T_{n}+I\right)}^{-1}\\ & T_{n}{\left(B_{n}-zI\right)}^{-1}{|}^{2})\\ \leq & \frac{1}{n^{2}}(Var\left\langle {\left({\underline{m}}_{n}\left(z\right)T_{n}+I\right)}^{-1}T_{n}{\left(B_{j}-zI\right)}^{-1}X_{\cdot j},X_{\cdot j}\right\rangle +\frac{C}{{\left(Imz\right)}^{2}})\\ \leq & K\left(z,k\right)\left(\frac{1}{n^{2}}\vee \frac{1}{n^{2k}}\right)\\ = & o(1), \end{array}$$

where *A* ∨ *B* = max{*A*, *B*}. Here we use Lemma 3.3 and the following facts from lemma 2.3 [13] and Eq (3.10) [18]; that is,

when $z=u+iv\in C^{+}$,

1.
${\left({\underline{m}}_{n}\left(z\right)T_{n}+I\right)}^{-1}\leq max\left(4\tau_{0}/Imz,\;2\right),\;{\left(B_{j}-zI\right)}^{-1}\leq 1/Imz$ , where the spectral norm ‖*T*_*n*_‖ can be controlled by a sufficiently large number *τ*_0_, i.e., ‖*T*_*n*_‖≤*τ*_0_, and this result is obtained by using a similar discussion from Silverstein and Bai [14].
2.
$|tr{\left({\underline{m}}_{n}\left(z\right)T_{n}+I\right)}^{-1}T_{n}{\left(B_{j}-zI\right)}^{-1}-tr{\left({\underline{m}}_{n}\left(z\right)T_{n}+I\right)}^{-1}T_{n}{\left(B_{n}-zI\right)}^{-1}|\leq C{\tau_{0}}^{2}/Imz$ .

Hence, when *n* → ∞, $\frac{1}{n}tr{\left(A_{n}-zI\right)}^{-1}-m_{n}\left(z\right)\to 0.$ The proof is completed.

**Lemma 2.5**. *Consider that the subsequence* $\left\{m_{n_{i}}\left(z\right)\right\}$ *of sequence* {*m*_*n*_(*z*)} *converges to* *m*. *Let* $\underline{m}=-\frac{1-c}{z}+cm$ *be the limit with respect to* ${\underline{m}}_{n_{i}}\left(z\right)$. *Then, the following function can be found*

$$\begin{array}{c} f\left(\tau \right)=\frac{1}{\underline{m}\tau +1}, \end{array}$$

*such that*

$$\begin{array}{c} f\left(\tau \right)-\frac{1}{{\underline{m}}_{n_{i}}\left(z\right)+1}\to 0. \end{array}$$

*Proof*. This lemma is to obtain that $F^{B_{n}}$ is tight. Define

$$\begin{array}{c} \delta =\underset{n}{\text{inf}}\;Im{\underline{m}}_{n}\left(z\right)\geq \underset{n}{\text{inf}}\;\int \frac{vdF^{{\underline{B}}_{n}\left(\lambda \right)}}{2\left(\lambda^{2}+u^{2}\right)+v^{2}}. \end{array}$$

By Lemma 2.8 [19], we get that *δ* is positive, a.s. So there exists *m* ∈ *D*_*c*_ such that $Im\underline{m}\geq \delta$, where $D_{c}=\left\{m\in C:-\frac{1-c}{z}+cm\in C^{+}\right\}$. If $m^{\prime }\in C^{+}$, and τ∈R, from the fact that

$$\begin{array}{c} |\frac{1}{m^{\prime }\tau +1}|\leq \frac{|m^{\prime }|}{Imm^{\prime }},\;\left|\frac{\tau }{m^{\prime }\tau +1}\right|\leq \frac{1}{Imm^{\prime }}, \end{array}$$

We obtain that function *f*(*τ*) is bounded and

$$\begin{array}{cc} |f\left(\tau \right)-\frac{1}{{\underline{m}}_{n_{i}}\left(z\right)+1}| & =|\frac{1}{\underline{m}\left(z\right)\tau +1}-\frac{1}{{\underline{m}}_{n_{i}}\left(z\right)\tau +1}|\\ & =|\frac{\tau (\underline{m}\left(z\right)-{\underline{m}}_{n_{i}}\left(z\right))}{\left({\underline{m}}_{n_{i}}\left(z\right)+1\right)\left(\underline{m}\left(z\right)+1\right)}|\\ & \leq \frac{1}{{\left(Im\underline{m}\right)}^{2}}|\underline{m}\left(z\right)||\underline{m}\left(z\right)-{\underline{m}}_{n_{i}}\left(z\right)|\\ & \leq \frac{1}{\delta^{2}}|\underline{m}\left(z\right)||\underline{m}\left(z\right)-{\underline{m}}_{n_{i}}\left(z\right)|. \end{array}$$

Then the lemma is proved.

Consequently, it can be obtained that

$$\begin{array}{c} \frac{1}{n}{\left(-z{\underline{m}}_{n_{i}}\left(z\right)T_{n}-zI\right)}^{-1}=\frac{1}{z}\int \frac{1}{{\underline{m}}_{n_{i}}\left(z\right)\tau +1}dF^{T_{n_{i}}}\left(\tau \right)\overset{n\to \infty }{\to }\frac{1}{z}\int \frac{1}{\underline{m}\tau +1}dH\left(\tau \right). \end{array}$$

Combined with Eq (4), we get that *m* satisfies Eq (5). Since *m* is the only solution satisfying Eq (5) [19], we have that *m*_*n*_(*z*) → *m*. By the results from Lemmas 2.1-2.5, the empirical spectral distribution $F^{B_{n}}$ of matrix *B*_*n*_ converges weakly to *F* almost surely, and the Stieltjes transformation of *F* satisfies Eq (5), and thus Theorem 1.1 is proved.

## 3. Appendix

In recent years, many studies have been conducted on the properties of random variables with isotropic distributions following log-concave distribution. We now introduce some related properties that not only help to understand the family of isotropic log-concave distributions, but also play an important role in the proofs. First, a result proved by Klartag [20] on large deviation is presented.

**Lemma 3.1**. *Let n* ≥ 1 *be a positive integer, and X be a random vector following isotropic log-concave distribution in* $R^{n}$, *then*

$$\begin{array}{c} P(|\frac{\left\|X\right\|}{\sqrt{n}}-1|\geq \frac{1}{n^{k}})\leq C\;\text{exp}\;\left(-n^{k}\right), \end{array}$$

*where C, k* > 0 *are constants*.

Based on this result, Pastur and Shcherbina [21] obtained the following concentration inequality about Euclidean norm.

**Lemma 3.2**. *Let n* ≥ 1 *be a positive integer, and X be a random vector following isotropic log-concave distribution in* $R^{n}$, *then*

$$\begin{array}{c} {Var(\|X\|}^{2})\leq C_{1}n^{-2k}, \end{array}$$

*where C*_1_, *k* > 0 *are constants*.

Pajor [15] used the above results to draw the following important conclusion, which is also a significant fact used in the proof.

**Lemma 3.3**. *Let M be a complex random matrix whose spectral norm is* ‖*M*‖ ≤ 1. *If* $X\in R^{n}$ (*or* $C^{n}$) *follows an isotropic log-concave distribution, then*

$$\begin{array}{c} Var\left\{\left\langle MX,X\right\rangle \right\}\leq bn^{-2k}, \end{array}$$

*where* *b*, *k* > 0 *are constants*.

## 4. Conclusions

This paper investigates the spectral properties of general *F* matrix $B_{n}=\frac{1}{N}X_{n}X_{n}^{*}T_{n}$. There has been a lot of work on the spectral properties of this matrix model, but the existing results always assume *X*_*n*_ being a matrix composed of *n* × *N* independent identically distributed elements. In this paper, we study that the column vector of *X*_*n*_ is a whole vector, not composed of unrelated independent components. Suppose that the column vectors of *X*_*n*_ follow isotropic log-concave distribution, we establish that the empirical spectral distribution of *B*_*n*_ converges to a non-random distribution probability *F* almost surely. Specifically, we prove that the Stieltjes transformation of *F* satisfies a deterministic form of equation and is the unique solution of the equation.
